# The interictal symptom burden in vestibular migraine—a condition in need of recognition

**DOI:** 10.3389/fneur.2026.1800235

**Published:** 2026-05-05

**Authors:** H. M. Rust, A. Palla

**Affiliations:** 1 Division of Vestibular Neurology, Department of Neurology, University Hospital Basel, Basel, Switzerland; 2 Faculty of Medicine, University of Basel, Basel, Switzerland; 3 Centre for Vestibular Neurology, Department of Brain Sciences, Imperial College London, London, United Kingdom; 4 Interdisciplinary Center for Neurological and Vestibular Disorders, Department of Neurology, University Hospital Zurich, Zurich, Switzerland; 5 Faculty of Medicine, University of Zurich, Zurich, Switzerland; 6 Bellevue Medical Group, Zurich, Switzerland

**Keywords:** chronic dizziness, interictal symptoms, migraine, persistent dizziness, persistent postural perceptual dizziness, threshold disorder, vestibular migraine

## Abstract

Vestibular migraine (VM) by current definition is an episodic vestibular disorder, yet many patients experience vestibular symptoms that persist beyond discrete attacks and extend into the interictal phase. Symptoms like head-motion intolerance, visually induced dizziness, positional vertigo, and increased susceptibility to motion sickness form an interictal symptom profile, which is often accompanied by migraine-associated sensory hypersensitivities. Clinical, psychophysical, vestibular laboratory, and ocular motor findings indicate altered central vestibular sensory and oculomotor networks, rather than a primary peripheral vestibular disorder. Longitudinal data indicate that interictal vestibular symptoms and ocular motor abnormalities are common and may increase over time, even when attack frequency decreases. These observations support a spectrum-based view of VM, in which episodic attacks and persistent interictal symptoms represent different clinical expressions within the same disorder. This narrative review summarizes current evidence on persistent interictal manifestations of VM and discusses implications for diagnosis and clinical decision-making in patients with episodic and chronic vestibular migraine phenotypes, using an operational definition of chronic vestibular migraine based on symptom frequency, for which preliminary diagnostic criteria are proposed.

## Introduction

Migraine is a variable disorder of brain function, modulated by intrinsic and environmental factors based on a genetically determined susceptibility. It is mainly characterized by episodic alterations in sensory processing and perceptual thresholds across multiple modalities, reflecting a transient dysfunction of brain networks rather than being merely a pain-related disorder (1, 2). In this context, vestibular symptoms such as dizziness and vertigo have long been recognized as part of the migraine spectrum (3). However, only in the last decades episodic attacks of vertigo and dizziness accompanied by migraine features have been widely recognized among clinicians as a nosological entity. Within the conceptual framework of an otherwise unexplained episodic vestibular disorder, the term “vestibular migraine” (VM) was established and in 2012 diagnostic criteria by an international expert group were published (4). Having almost exclusively been referred to as an episodic disorder, the matter of chronic, persistent sensitivity to head movements, otherwise apparent dizziness and abnormal vestibular function in many patients with VM outside VM or migraine attacks (“interictal phase”) has received only little attention. Those symptoms have been attributed to persistent interictal abnormalities of brain function and have been discussed in a figurative sense by some authors within the context of a chronic variant of VM (5–8). In this review, we provide an overview of selected studies and our own clinical observations on persistent symptoms and clinical signs occurring during the interictal phase in patients diagnosed with VM. We introduce operational definitions for interictal vestibular symptoms, persistent (chronic) vestibular symptoms, and propose a preliminary diagnostic framework intended to stimulate prospective validation.

## Terminology

Given the heterogeneity of the terminology across the literature, we apply standardized operational definitions throughout this review to ensure internal consistency and comparability between studies.

In this narrative review, the term “interictal phase” refers to the time period outside of clearly defined vestibular migraine attacks, as defined by the Bárány Society diagnostic criteria (4, 9), i.e., attacks lasting ≥5 min and ≤72 h, irrespective of whether patients are completely asymptomatic or experience persistent baseline symptoms.

We use the term “interictal vestibular symptoms” to denote vestibular symptoms (dizziness or vertigo) that occur during this interictal phase. These symptoms typically persist for at least 72 h beyond a clearly defined attack, but may also be shorter than 5 min.

The term “persistent (chronic) vestibular symptoms” is used descriptively for interictal vestibular symptoms that last for ≥3 months without complete remission, in line with Bárány Society diagnostic frameworks for chronic vestibular disorders (i.e., e.g., persistent postural-perceptual dizziness).

Where original studies use different operational definitions (e.g., “chronic dizziness” defined as ≥6 months, or “daily symptoms”), these are reported verbatim in quotation marks to preserve comparability with the source literature.

## Search methods

This review was conducted as a narrative review; accordingly, the search strategy was targeted rather than systematic, and no PRISMA flowchart was generated. A literature search was performed in PubMed and Google Scholar between March and December 2025 (final retrieval dates: 17–19th December 2025) to identify publications addressing persistent interictal symptoms in VM. The PubMed search covered the period from 2015 onwards; earlier key publications were additionally identified through reference list screening. Only publications in English were considered.

Search terms included the MeSH heading “Vestibular Migraine” and its entry terms (“migrainous vertigo,” “migraine-associated vertigo,” “migraine-related vestibulopathy”), as well as corresponding free-text terms applied to title and abstract fields. These were combined using Boolean operators (AND/OR); for example: “vestibular migraine”[Title/Abstract] AND (“clinical” OR “clinical features” OR “phenotype” OR “vertigo” OR “dizziness” OR “migrainous vertigo” OR “migraine associated vertigo” OR “migraine associated dizziness” OR “migraine related vestibulopathy”).

The combined database search initially identified 188 records. After removal of duplicates and screening of titles and abstracts by AP and HMR, full-text review was performed where necessary, resulting in 49 eligible publications. Reference lists of included publications were hand-searched to identify additional relevant studies, and further articles were incorporated based on the authors’ clinical expertise.

## Interictal symptoms and clinical signs in vestibular migraine

### Temporal characteristics of vestibular symptoms

Temporal aspects in this section refer to interictal symptom persistence beyond discrete VM attacks, acknowledging that boundaries between ictal and interictal phases are not uniformly defined across studies. In VM, episodes of dizziness, vertigo, and head-motion sensitivity are typically accompanied by headache or other migraine features, usually lasting for hours up to 1 day. Between attacks, patients commonly do not report vestibular symptoms. However, a substantial proportion of patients report persistent vestibular symptoms after a VM attack that continue for days, weeks, or even months (10–12). Cutrer and Baloh mention in their review of 91 patients with “migraine-associated dizziness” a subset of patients, not otherwise specified, who reported a dizzy sensation (“motion-sickness-like sensation which they described with terms such as swimming or rocking inside their head but without the feeling that they were in motion in relation to their environment”) occurring in episodes of gradual onset and tending to persist for days to weeks (11). In Waterston’s case series on “chronic migrainous vertigo”, in whom he attributed persistent vestibular symptoms to be migraine-associated, all 16 subjects had a history of chronic dizziness for at least 6 months prior to medical assessment. Many subjects experienced daily symptoms, whereas others suffered from episodes lasting weeks to months, interrupted only by brief symptom-free intervals (8). Two retrospective studies found long-lasting interictal vestibular symptoms (“chronic vestibular symptoms”/“persistent dizziness” in the original reports) to be present in half of the patients (3, 10). In a recent study, dizziness between VM attacks was reported in 36.6% of 415 patients (47), which increased in a follow-up study to 67% (13, 47). Interictal vestibular symptoms were reported by 24.8% of subjects in a prospective study in 101 VM patients, while daily vestibular symptoms were reported by 10% (14), which is consistent with the percentage reported by Huang et al. (12) and corresponds to a frequency compatible with a chronic vestibular migraine phenotype as defined in this review. In the first study addressing the subject of persistent dizziness in VM, Chae et al. applied the criteria for episodic and chronic migraine of the International Classification of Headache Disorders (ICHD) to patients with a diagnosis of VM and defined definite and probable “chronic VM” when vestibular symptoms were present ≥15 days per month, which is conceptually in line with the operational definition of chronic vestibular migraine used in this review. In the subgroup of definite chronic VM, almost 80% reported constant vestibular symptoms (15).

## Head-motion intolerance

Persistent sensitivity to head motion in patients with VM has been named “head motion induced dizziness” (4), “head motion intolerance” (16), “head motion-induced vertigo” (17), “dizziness induced by head movement” (18) and “head motion dizziness” (3). Within the context of VM, those terms likely describe what was defined as “head motion vertigo (or dizziness)”, i.e., “vertigo (or dizziness) occurring only during head motion (that is, time-locked to the head movement)” (19). This condition should not be confused with the more severe intolerance of head movements in the acute vestibular syndrome (20, 50). Typically, VM patients report no dizziness or only a slight dizzy feeling when they are at rest and not moving.

Using the term “head motion intolerance”, Salhofer et al. described it as the most prominent specific feature of “migraine vertigo” in their prospective diary study (21). In a retrospective study in 131 patients, interictal “head-motion dizziness” was recorded in 65.6% (3). Interictal dizziness during self-motion or motion sickness was present in 69% of patients in a follow-up assessment of clinical symptoms and vestibulo-cochlear findings in 61 patients with VM after a median follow-up of 9 years (16). Motion was reported to be the most common trigger for vestibular symptoms in patients who were regarded as having “chronic VM” (94%) (15).

## Visually induced dizziness

“Visually-induced dizziness” was defined by Bisdorff et al. as “vertigo (or dizziness) triggered by a complex, distorted, large-field, or moving visual stimulus, including the relative motion of the visual surround associated with body movement” (19, 22). Already in 1981, Kuritzky et al. reported from their prospective questionnaire study on vestibular symptoms in patients with migraine that “visual vertigo,” which they referred to as “while looking at spinning objects”, occurred in their “classical migraine group” in 61% (23). In an experimental study on visual–vestibular interaction in VM, a considerable proportion of study participants who were classified as having VM reported symptoms of “feeling unwell” and being “symptomatic-dizzy” after having been exposed to optokinetic stimulation, compared to controls (24).

Visually induced dizziness was reported to be present interictally in a retrospective study by Beh et al. in 131 patients in almost 90% of patients (3). In another study, this feature was recorded in 54% of 61 subjects (16). In a prospective study in 101 patients, 71.3% reported induction and/or aggravation of vestibular symptoms when exposed to “moving visual stimuli (gazing at moving vehicles, people, movie/TV/computer screen)” and 47.5% reported the same when they were exposed to “visually complex or busy environments (shopping malls, supermarkets, crowded areas)” (14).

### Positional vertigo

“Positional vertigo (or dizziness) is vertigo (or dizziness) triggered by and occurring after a change of head position in space relative to gravity” (19). Interictal positional vertigo was reported by 20.2% out of 203 patients in a cross-sectional questionnaire study (13). In a population of 61 subjects with VM, 39% had positional vertigo, which increased to 80% after a median follow-up of 9 years (16). In a cross-sectional study in 415 patients with VM, 21.7% of patients described positional vertigo attacks during the ictal/interictal phase (otherwise not further specified) (47). There is considerable symptom overlap between VM and benign paroxysmal positional vertigo (BPPV) and VM mimicking BPPV (25).

### Photophobia and phonophobia

In a population-based study in 871 people with migraine, no significant association could be found for photophobia and phonophobia with migraine with dizziness and/or vertigo (49). In a retrospective study in 131 patients with VM, photophobia was recorded in 10.7% and phonophobia in 2.3% interictally (3). In a study on different subtypes of VM, with chronic VM being a defined entity with vestibular symptoms present on more than 15 days per month, photophobia was present in 77% and phonophobia in 82% of those patients with chronic VM (15). In a cross-sectional study in 366 patients, the presence of ictal photophobia correlated significantly with the level of interictal photosensitivity (26). Rates of photophobia were similar in patients with VM, in those with chronic migraine who fulfilled the Bárány Society criteria for VM, and in subjects with chronic migraine without vestibular symptoms. Photophobia was found not to be specific for the aforementioned groups (27).

### Cutaneous allodynia

From a population-based study (871 patients with definite migraine), Akdal et al. reported that allodynia is significantly more prevalent in those migraineurs experiencing vertigo and dizziness than in those who do not (49). Toriyama et al. also reported a significant association of allodynia with VM and found no significant association with migraine without vestibular symptoms in a study in 163 patients (28). In a prospective study in 27 patients with VM and 40 patients with chronic migraine, cutaneous allodynia was most prevalent and most severe in those patients with chronic migraine who also fulfilled the Bárány Society criteria for VM (27).

### Auditory symptoms

From a retrospective study in 166 patients with VM, mainly mild low-frequency hearing loss (125, 250, and 500 Hz) was detected during tone audiometry (PTA) tests in 21% (bilateral hearing loss in 3.6% and unilateral hearing loss in 17.5%). At a follow-up PTA assessment after 1 month, hearing loss in the low to medium frequency range had reversed in all patients. In a cross-sectional study including 51 patients with definite or probable VM, 75% of patients reported tinnitus, which was not associated with hearing loss (45). In a cross-sectional follow-up study in 203 patients with at least 4 years of follow-up, cochlear symptoms were reported to rise from 15% initially to 49%, with 18% having developed mild bilateral sensorineural hearing loss (13). Even higher rates on follow-up in a study in 61 patients (median follow-up 9 years) were reported to be present interictally by Radtke et al., rising from 26 to 77% (16).

### Motion sickness

People with VM are usually highly susceptible to motion sickness (29–31). In a retrospective study in 166 patients with VM, motion sickness was reported by 67.5% (32). Neff et al. reported a history of motion sickness in 51% of patients with VM (33). A history of motion sickness is significantly more frequent in patients with VM than in controls (34). Motion sickness susceptibility is also more frequent in VM than in migraine-only patients: using a single screening question, Akdal et al. found that inability to read while travelling in a car—a marker of motion sickness susceptibility—was reported by 85.6% of patients with VM compared with 50% of migraine-only patients (35). When compared with other vestibular disorders, symptoms of motion sickness while reading as a passenger were also significantly more frequent in patients with VM (91.2%) than in those with persistent postural–perceptual dizziness (46.7%) or other vestibular diagnoses (23.5%) (46). Finally, a history of motion sickness was present in 70% of patients considered as having chronic VM by Chae et al. (15).

### Interictal ocular motor abnormalities

In a retrospective study in 90 patients with episodic vertigo and dizziness attributed to migraine, 65.6% showed ocular motor abnormalities in the interictal phase. Spontaneous nystagmus without fixation was present in 11%, gaze-evoked nystagmus in 27%, moderate positional nystagmus in 11%, vertical and horizontal smooth pursuit abnormalities in 48 and 22%, respectively. Dissociated gaze-evoked nystagmus was present in 9%, while downbeat/upbeat nystagmus was recorded in 9% (51). In a study in 415 patients, none had spontaneous nystagmus in the interictal phase (47). In a retrospective study in 131 patients, none had spontaneous nystagmus in primary position, but 9.9% did so when fixation was removed. Gaze-evoked nystagmus was present in 3.8%. Central positional nystagmus (without associated vertigo, latency, or fatigability, and persisting as long as the head was maintained in the provoking position) was present in 18.5%, most commonly triggered by right and left Dix–Hallpike manoeuvres (3). In a cross-sectional study in 415 patients with VM, typical posterior canal BPPV-type positional nystagmus was present during Dix–Hallpike testing in 12.3% of patients (47). Teggi et al. reported bipositional, long-lasting (more than 1 min), non-paroxysmal, apogeotropic nystagmus interictally in 23.5% of 34 VM patients in their study (44).

From a study in 203 patients who were followed up for at least 4 years, the percentage of interictal ocular motor abnormalities was reported to increase from 16% initially to 41% at follow-up, with positional nystagmus being the most frequent finding, detected in 28% (13). Ocular motor abnormalities were observed to increase from 15 to 41% in another follow-up study in 61 patients (16). The most frequent finding in this study was positional nystagmus in 28% (definite central positional nystagmus in 18%) (16).

## Discussion

### Summary of main findings

VM is commonly framed as an episodic disorder; however, available evidence indicates that a considerable subpopulation of patients—from approximately 10% in prospective cohorts to more than 50% in retrospective and longitudinal studies—experience persistent vestibular symptoms beyond discrete attacks, extending into the interictal phase [e.g., (3, 12–14, 47)]. Across studies, interictal symptom burden spans a continuum from transient post-attack persistence to long-lasting symptoms over weeks to months, or near-continuous baseline dizziness or vertigo [e.g., (8, 11, 13)]. The interictal phenotype is dominated by heightened sensitivity to self-motion and visual motion, frequently accompanied by positional triggers and motion sickness (3, 14–16), a pattern consistently reported across independent cohorts and suggestive of persistent abnormalities in vestibular–visual processing. Objective interictal signs, particularly ocular motor abnormalities and positional nystagmus, have been documented in a substantial proportion of patients (3, 13, 16, 51) and support the presence of altered brain function beyond VM attacks. Longitudinal studies demonstrate that interictal dizziness and ocular motor abnormalities may increase over time (13, 16), even as the frequency or severity of VM attacks decreases (13), suggesting that the interictal symptom burden may evolve independently from classical attack frequency. However, these longitudinal patterns need to be interpreted with caution, as key potential confounders—including treatment changes, comorbid conditions and migraine chronification—were not consistently controlled for across cohorts. Despite these limitations, the available evidence suggests that interictal symptom burden reflects an evolving dimension of disease activity rather than a simple by-product of attack frequency.

In analogy to migraine, VM may therefore be conceptualized as a threshold disorder in which altered sensory processing and fluctuating susceptibility to vestibular, visual, and somatosensory inputs are associated with both episodic attacks and persistent baseline symptoms (2). Within this framework, interictal vestibular symptoms represent an integral expression of disorder activity rather than an epiphenomenon confined to distinct attacks.

Against this clinical background of persistent interictal symptoms, experimental evidence indicates that vestibular hypersensitivity in VM predominantly is more consistent with altered central processing rather than peripheral vestibular dysfunction. Quantitative psychophysical studies consistently demonstrate reduced perceptual thresholds for passive self-motion in interictal VM patients compared with healthy controls and migraine patients without vestibular symptoms. Importantly, these reductions are confined to motion paradigms requiring central integration of semicircular canal and otolith signals, such as dynamic roll tilt, whereas thresholds for isolated canal or otolith stimulation remain normal (36, 37). This selective pattern argues against generalized peripheral vestibular hypersensitivity and is compatible with dysfunction at the level of higher-order vestibular integration.

Further evidence for a central perceptual mechanism comes from rotary chair experiments. In interictal VM patients, yaw rotation perception thresholds are lower and susceptibility to motion sickness is increased, while vestibulo-ocular reflex thresholds and slow-phase velocities remain comparable to controls (38). The dissociation between enhanced perceptual sensitivity and normal reflexive vestibular responses reinforces the concept that vestibular hypersensitivity in VM primarily is more consistent with altered central perceptual processing rather than abnormalities of basic vestibular reflex pathways.

Clinical data from large cross-sectional studies extend these experimental findings by placing persistent vestibular symptoms within the broader framework of migraine-related sensory sensitization. Vestibular symptoms are strongly associated with interictal multisensory hypersensitivity and overall migraine burden, with a graded relationship between the number of interictal sensory hypersensitivities, headache frequency, and both the prevalence and severity of dizziness and vertigo (39). Together, these associations suggest that persistent vestibular symptoms scale with migraine burden and symptom frequency rather than reflecting a distinct vestibular disorder. Persistent interictal motion sensitivity and baseline dizziness may therefore be considered integral manifestations of VM in a subset of patients.

Further support for this frequency-based conceptualization is provided by prospective ecological momentary assessment data. Using daily symptom sampling, Saroya et al. showed that VM patients experience vestibular symptoms on most days, with a mean of fewer than four symptom-free days per month, and that approximately 60% meet a threshold of ≥15 days per month with moderate to severe dizziness (40). Analogous to the distinction between episodic and chronic migraine, the term “chronic VM” may thus be applied descriptively to patients characterized by sustained vestibular symptom frequency rather than discrete attack patterns.

### Vestibular migraine: a spectrum disorder

In light of an ongoing debate among headache and vestibular specialists, VM may be best understood as an individual-specific clinical presentation of migraine in which vestibular symptoms constitute the predominant manifestation rather than a strictly separate diagnostic entity. Cross-sectional data demonstrate substantial clinical overlap between patients fulfilling diagnostic criteria for VM and those with migraine-associated vestibular symptoms not meeting formal criteria, with no meaningful differences in core clinical features and a shared high prevalence of cutaneous allodynia as a marker of central sensitization (28). Further evidence indicates that sensory hypersensitivity in VM extends beyond vestibular symptoms: in a study of 366 patients, ictal photophobia was positively associated with increased interictal photosensitivity and visually triggered dizziness, suggesting altered visual sensory that may extend outside acute attacks (26). These observations support a spectrum-based rather than categorical distinction between VM and other migraine presentations with vestibular involvement (12).

From this perspective, important clinical implications arise for the interpretation of persistent vestibular symptoms. In patients with long-standing, near-continuous dizziness and motion sensitivity, a strict categorical separation between interictal VM phenomena and persistent postural–perceptual dizziness (PPPD) may be inherently challenging. This diagnostic overlap parallels the situation in chronic migraine, where high symptom frequency and variability limit the reliable distinction of individual headache phenotypes and symptom frequency becomes the defining clinical criterion according to the International Classification of Headache Disorders, 3rd edition (ICHD-3). By analogy, rigid attempts to separate PPPD from pronounced interictal VM-related manifestations may be of limited clinical utility. Nevertheless, the studies reviewed here suggest that certain clinical features can support the differential diagnosis at the bedside. For clinical purposes we have included a table with cardinal features of the respective entities PPPD and chronic vestibular migraine in order to allow a distinction at the bed side.

**Table 1:** 

	PPPD	CVM
Migraine link	Not mandatory	Mandatory
Vestibular symptoms exacerbated on upright posture	Mandatory	Not mandatory
Head-motion intolerance	No typical feature	Typical feature
Sensory hypersensitivity	No typical feature	Typically, heightened sensitivity to exteroceptive input (e.g., photophobia, phonophobia)
Motion sickness susceptibility	No typical feature	Typical feature
Oculomotor findings	No typical feature; abnormal findings suggest comorbidity	Possible abnormalities, typically low-frequent positional nystagmus
Postural avoidance behaviour	Prominent feature; may reach phobic dimensions	No typical feature

Visually induced dizziness, while considered a core feature of PPPD (41), is also highly prevalent in VM and therefore lacks sufficient specificity when considered in isolation. By contrast, the presence of cutaneous allodynia—reflecting central sensory sensitization—and the presence of additional migraine-associated sensory features—including photophobia, phonophobia, or auditory symptoms may provide supportive evidence for a migraine-related mechanism in patients presenting with persistent dizziness and motion sensitivity (26, 32, 48). Overall, this perspective underscores that persistent vestibular symptoms should not be reflexively attributed to a separate functional vestibular disorder. Instead, they may represent a migraine-related phenotype in a substantial subset of patients, highlighting the need for a nuanced, spectrum-based clinical assessment rather than rigid diagnostic categorization.

### Quality and limitations of current evidence

One of the main issues when trying to estimate the impact or the intensity of persistent symptoms is that interictal symptoms are not clearly defined in most studies, nor is their intensity or functional impact systematically assessed using standardized, validated outcome measures. Although Beh et al. state that interictal visually induced dizziness and head motion dizziness were the predominant causes of impaired job performance and diminished participation in household and social activities (3), these aspects are not captured by standardized severity metrics in most cohorts. This limitation is compounded by the predominance of retrospective study designs, which are inherently susceptible to recall bias. As most studies were conducted at tertiary centres for neuro-otology, the samples may be biased towards more severe cases, with longer time from symptom onset and more pronounced degrees of persistent vestibular symptoms, thereby limiting the generalisability of findings to community-based populations.

In the studies reviewed, some of the descriptions of symptoms were not based on otherwise defined definitions, e.g., the term “visually-induced dizziness” (19). Related terms such as interictal, persistent or chronic vestibular symptoms are used heterogeneously across studies, further reducing comparability between cohorts. In this review, we therefore apply explicit operational definitions for interictal vestibular symptoms, persistent vestibular symptoms, and chronic vestibular migraine (see Terminology), and we reproduce the original wording of individual studies in quotation marks where their usage differs from ours.

Motion sickness represents a particular challenge in this context, as most studies assess it as a lifetime susceptibility or historical feature rather than explicitly during the interictal phase. As a result, available data do not allow a strict temporal classification as an interictal symptom; instead, motion sickness susceptibility in migraine and VM is typically conceptualized as a relatively stable, person-specific vulnerability to motion-related sensory conflict that can persist between attacks [e.g., (31)].

In studies assessing interictal oculomotor abnormalities, velocities of slow-phase eye movements were not clearly defined. From our empirical observations, in most people up to the age of over 65 there are typically no clinically relevant nystagmoid eye movements when assessed with Frenzel goggles. However, given that migraine affects approximately 15% of the population (42), earlier studies on oculomotor abnormalities in “normal” subjects have not taken this fact into account and might therefore not be representative in terms of what can be regarded as “normal” or what might be an “interictal” abnormality in an otherwise healthy subject.

Longitudinal studies are further limited by variability in follow-up duration, differential attrition, comorbid conditions, and the potential influence of treatment changes and migraine chronification on symptom trajectories, which were not systematically accounted for across studies. These factors may influence both the perception of dizziness and the likelihood of detecting interictal ocular motor abnormalities and could partly account for the observed increase in interictal symptom burden over time.

### Proposed diagnostic framework for chronic vestibular migraine

Based on the evidence reviewed, and by analogy with the distinction between episodic and chronic migraine in the ICHD-3 (43), we propose a preliminary diagnostic framework for chronic vestibular migraine (CVM). The format of the classification is modelled on the International Classification of Headache Disorders and the Bárány Society vestibular migraine criteria (4, 9), and uses the same frequency threshold (vestibular symptoms on ≥15 days per month for ≥3 months) specified in the Terminology section.

The only prospective study to date to operationalise diagnostic criteria for a chronic VM subtype is that of Chae et al. (15), with whom the present framework converges in its two most fundamental design choices: a definite/probable distinction modelled on the Bárány Society classification. The present proposal extends the Chae et al. framework in three respects: it adds a minimum duration requirement of 3 months to exclude transient high-frequency phases; it introduces a cumulative daily symptom burden threshold (≥4 h on symptomatic days) to distinguish clinically significant chronification from frequent brief episodes; and it operationalises an interictal vestibular profile criterion capturing head-motion dizziness, visually induced dizziness, and motion sickness susceptibility (3, 14). It also departs from Chae et al. in treating the chronic phenotype as an entry point in its own right, rather than requiring prior fulfilment of episodic Bárány criteria—consistent with the observation that many patients with chronic VM never present with discrete attacks of qualifying duration (15).

Consistent with clinical observations and the ecological momentary assessment data of Saroya et al. (40), the dominant interictal symptom in this patient population is dizziness. This pattern of near-continuous dizziness is consistent with the interictal symptom profile described in this review –including among others head-motion dizziness, visually induced dizziness, and increased motion sickness susceptibility (3, 14). It underscores that vestibular symptoms in this chronic phenotype often manifest as a persistent sensory hypersensitivity rather than discrete attacks (36, 37, 39). In analogy with chronic migraine, in which the characteristic headache progressively takes on features of tension-type headache as the dominant daily symptom, we speculate that persistent non-specific dizziness may represent the vestibular equivalent of this transformation—the chronic background noise of a disorder that, in its episodic form, presents with discrete vestibular attacks.

The proposed diagnostic criteria for CVM (Box 2) are intended to stimulate prospective validation and should not be interpreted as consensus criteria. Their formal establishment will require prospective studies using statistical approaches to determine optimal feature combinations and the clinical utility of the definite/probable distinction. Formal evaluation by the Bárány Society and relevant headache societies will be an essential next step.

**Table 2:** 

Chronic vestibular migraine A. Temporal criterion Vestibular symptoms (vertigo or dizziness, at least moderate intensity) on ≥15 days per month for 3 months or more B. Vestibular profile At least 2 of the following features (ictal or interictal): ◦ Head motion dizziness/vertigo, time locked to head movement ◦ Visually induced dizziness/vertigo triggered by moving visual stimuli or complex visual environments ◦ Positional dizziness/vertigo triggered by head position changes relative to gravity ◦ Increased motion sickness susceptibility ◦ Persistent migraine associated sensory hypersensitivity (interictal photophobia, phonophobia and/or cutaneous allodynia) C. Migraine–vestibular link Current or previous diagnosis of definite or probable vestibular migraine (Bárány), or a clinical history highly compatible with vestibular migraine (recurrent vestibular episodes with migrainous features) And current or previous history of migraine with or without aura according to ICDH-3 D. Migraine association Vestibular symptoms are at least occasionally temporally associated with chronic migrainous features (migraine-like or tension-type-like headache and/or nausea and/or vomiting, photophobia/phonophobia and/or aggravation by or causing avoidance of routine physical activity) Migrainous features do not need to be present every day; in near continuous courses, diagnosis is based on the overall history and pattern of frequent vestibular symptoms within a migraine/vestibular migraine disorder, not on day-by-day attribution E. Exclusion Symptoms are not better accounted for by another Barany vestibular diagnosis or by another ICHD 3 diagnosis Diagnostic levels *(proposed)* Definite chronic vestibular migraine: criteria A + B + C + D fulfilled Probable chronic vestibular migraine: criteria A + B + (C or D) fulfilled

## Conclusion

This review demonstrates that persistent interictal vestibular symptoms and ocular motor abnormalities occur far more frequently than traditional diagnostic criteria for episodic VM would suggest. In many patients with a diagnosis of VM, symptom presentation does not conform to a purely episodic pattern but reflects a continuum of persistent and episodic manifestations, consistent with a spectrum-based view of the disorder. Available evidence indicates that persistent vestibular symptoms and interictal ocular motor abnormalities may not be incidental comorbidities but could represent core features of VM in a substantial subset of patients. Recognizing this chronic end of the spectrum is important for attributing these often persistent heterogeneous symptoms to a migraine related disorder. To provide a framework for clinical recognition and future research, we propose preliminary diagnostic criteria for chronic vestibular migraine, modelled on the ICHD-3 and Bárány Society classifications, which require prospective validation.
